# Patterns of Exchange of Multiplying Onion (*Allium cepa* L. Aggregatum-Group) in Fennoscandian Home Gardens

**DOI:** 10.1007/s12231-018-9426-2

**Published:** 2018-10-22

**Authors:** Matti W. Leino, Svein Ø Solberg, Hanna Maja Tunset, Jesper Fogelholm, Else-Marie Karlsson Strese, Jenny Hagenblad

**Affiliations:** 1 The Archaeological Research Laboratory, Department of Archaeology and Classical Studies, Stockholm University, SE-106 91 Stockholm, Sweden; 2 IFM-Biology, Linköping University, SE-581 83 Linköping, Sweden; 3 Nordic Genetic Resource Center, SE-230 53 Alnarp, Sweden; 4 Inland Norway University of Applied Sciences, Postboks 400, 2418 Elverum, Norway; 5 Department of Biology, Norwegian University of Science and Technology, 7491 Trondheim, Norway; 6 Swedish Buseum of Agriculture, Nordic Museum, 27820, SE-115 93 Stockholm, Sweden

**Keywords:** Aggregating onion, shallot, potato onion, on-farm conservation, SSRs

## Abstract

Multiplying onion (*Allium cepa* L. Aggregatum-Group), commonly known as shallot or potato onion, has a long tradition of cultivation in Fennoscandian home gardens. During the last decades, more than 80 accessions, maintained as vegetatively propagated clones, have been gathered from home gardens in all Fennoscandian countries. A genetic analysis showed regional patterns of accessions belonging to the same genetic group. However, accessions belonging to the same genetic group could originate in any of the countries. These results suggested both short- and long-distance exchange of set onions, which was confirmed by several survey responses. Some of the most common genetic groups also resembled different modern varieties. The morphological characterization illustrated that most characters were strongly influenced by environment and set onion properties. The only reliably scorable trait was bulb skin color. Neither our morphological nor genetic results support a division between potato onions and shallots. Instead, naming seems to follow linguistic traditions. An ethnobotanical survey tells of the Fennoscandian multiplying onions as being a crop with reliable harvest, excellent storage ability, and good taste. An increased cultivation of this material on both household and commercial scale should be possible.

**Electronic supplementary material:**

The online version of this article (10.1007/s12231-018-9426-2) contains supplementary material, which is available to authorized users.

## Introduction

Countries with highly industrialized agriculture and horticulture are often considered effectively void of landraces still being in cultivation. However, an often neglected source of biodiversity is home gardens where heirloom vegetable crops sometimes have been preserved for generations (Galluzzi et al. 2010). The vegetables preserved in home gardens often have different characteristics from commercial ones, and traits such as taste, easy propagation, and storage capacity are more valued than solely high yield. In the Fennoscandian countries (Denmark, Sweden, Norway, and Finland), the typical *Allium* species commercially cultivated are bulb onion (*Allium cepa* L. Common onion-Group) and leek (*Allium ampeloprasum* L.), both propagated from seed of hybrid varieties. In contrast, home gardeners often use indigenous multiplying (aggregating) onion, known as shallot or potato onion (*Allium cepa* L. Aggregatum-Group). These onions are clonally propagated, and they rarely flower and set seed under Fennoscandian climatic conditions. The name potato onion probably stems from the similarity to potato propagation. Cloves of the onions are planted in the spring, and in early autumn, a cluster of, normally around ten, onions can be harvested. The onions grow fast and are hardy even in northern Scandinavia and are known for having excellent storage capacity (Nygårds and Leino 2013).

Onions (here in the sense of all edible *Allium* species) are mentioned already in the earliest written sources concerning Scandinavia. For example, there are reports of Vikings bringing onions on their ships during long-distance sea travel during the 10th century (Robertson 1978). Many lines of evidence suggest that multiplying onion may have been the dominating *Allium* species historically in the Nordic countries (Leino and Hagenblad 2014). In the early 20th century, potato onion and shallot, usually without cultivar names, were commercially available in Denmark and Sweden (Börjeson 2015). In Finland, set onions produced in Russia were sold (Lundén 1920). It is not known whether the heirloom multiplying onions still cultivated in the 21st century are the descendants of 20th century introductions or remnants of the older cultivation tradition.

Multiplying onions have historically, as well as today, been classified as either potato onions or shallots. Bulb shape, skin color, taste, and storage capacity are often mentioned as discriminating characteristics. A clear distinction between shallot and potato onion based on such morphological characters is, however, difficult to make and sources are contradictive (Fritsch and Friesen 2002; Hanelt 1990; Rabinowitch and Kamenetsky 2002). The present Swedish standard for nomenclature of cultivated plants also differentiates between potato onion and shallot, but admits that the borders are unclear (Aldén et al. 2009). Whether shallot and potato onion form genetically differentiated groups is not known.

The Nordic gene bank (NordGen), as well as gardening NGOs and national crop biodiversity programs in the Nordic countries, has been gathering locally cultivated vegetables during the last decades (Nygårds and Leino 2013; Osara 1987). At present, more than 80 accessions of multiplying onion have been collected. Maintaining these as clones in ex situ field gene banks, however, is expensive, and identification of putative duplicate accessions as well as accessions being unique would be valuable for reducing the collection without loss of diversity. Furthermore, information on the cultural background of origin and cultivation of this crop is important for a small-scale commercialization that in turn would contribute also to *in situ* preservation of the crop.

Here, we present an analysis of the genetic diversity of this material, together with morphological characterizations and an ethnobotanical survey aimed to address the following questions: (1) How diverse are accessions of Fennoscandian multiplying onion?, (2) What are the geographical patterns of seed (set onion) exchange?, (3) What is the relationship to commercial clones?, and (4) Can genetic or morphological differences between potato onion and shallot be identified?

## Materials and Methods

### Plant Material and Morphological Characterization

A total of 84 locally cultivated multiplying onion accessions from Fennoscandia—Denmark, Finland, Norway, and Sweden—were studied. The accessions were obtained from NordGen, the Nordic Genetic Resource Center (accessions  with prefix NGB), or from the Swedish National Program for Diversity of Cultivated Plants (accessions with prefix SWE). As a contrasting material for the genetic analysis, six local accessions from Georgia and eight modern varieties were included. These accessions were obtained from The Leibniz Institute of Plant Genetics and Crop Plant Research (IPK), Gatersleben, Germany (accessions with prefix ALL), or purchased commercially. Materials are summarized in Table 1 and the full list of accessions is given in Electronic Supplementary Material 1 (ESM). Different sets of accessions (the Fennoscandian material) have previously been test cultivated in Landvik, Norway (58° 20′ N, 08° 31′ E) during 2001; in Aarslev, Denmark (55° 18′ N, 10° 27′ E) during 2002; in Alnarp, Sweden (55° 39′ N, 13° 04′ E) during 2009–2011; in Röttle, Sweden (57° 59′ N, 14° 25′ E) during 2011; or in Julita, Sweden (59° 08′ N, 16° 02′ E) during 2013–2014. They were scored for relevant morphological characters according to IPGRI descriptors for *Allium* spp. (IPGRI, ECP/GR, AVRDC 2001). Data from these test cultivations are assembled (ESM 1).

**Table 1:** groups of accessions used in the study and genetic diversity within groups.

Group	No. of accessions	Genetic diversity within group (Nei’s *h*)
Denmark, landraces	24	0.48
Finland, landraces	14	0.58
Georgia, landraces	6	0.23
Norway, landraces	17	0.58
Sweden, landraces	29	0.52
Modern varieties	8	0.66
Total	98	

### Genetic Analysis

Green shoots or the innermost layers of onion bulbs (< 100 mg fresh weight) were used for DNA extraction. The extractions were performed using either the E.Z.N.A Plant DNA kit from Omega Biotek (Omega Biotek, GA, USA) or the DNeasy plant mini kit from Qiagen (Qiagen AB, Germany) in accordance with the manufacturers’ instructions. Genotyping was performed using 12 microsatellite markers originally designed for bulb onion (*Allium cepa* Common onion-Group) (Fischer and Bachmann 2000). Table 2 presents the studied markers, primer sequences, and temperatures. The PCRs were performed in a total reaction volume of 20 μl containing 0.2 μM forward primer fluorescently labeled with either FAM or HEX, 0.2 μM reverse primer, 0.25 μM each of dNTPs, 60–100 ng template DNA, and 0.5 U Dream Taq DNA polymerase and supplied buffer (Fermentas, Hanover, MD). The PCR cycling parameters were denaturation for 3 min at 94 °C followed by 35 cycles of 15 s of denaturation at 94 °C, 30 s annealing at 52–62 °C, 30 s of extension at 72 °C, and final elongation step of 72 °C for 10 min. PCR fragment lengths were analyzed by capillary gel electrophoresis and confocal laser scanning using an ABI 31030x1 genetic analyzer. Alleles were identified using the software Geneious version 6.1.8 (Kearse et al. 2012).

**Table 2:** markers, primer sequences, and corresponding annealing temperatures.

Marker	Primer sequence Forward	Primer sequence Reverse	Annealing temperature (°C)
AMS02	GCA TTA ACT ATC TAA AAC ATT G	CCA TCA ACT CAT AAC AGG T	53
AMS06	GGT GCA TAG GGT CTC ATC TG	ATT GAT TGT TTG TTT GGA TGT G	56
AMS08	GCC ACG ATG TTG AGA TTT CG	CCC GAA TAT CCC ACC AGT TC	55
AMS12	AAT GTT GCT TTC TTT AGA TGT TG	TGC AAA ATT ACA AGC AAA CTG	58
AMS14	CCC CTG AGT AAA TTC AAA ATC C	TCC TTA GTA TAA TTT CGG GGT AAC	62
AMS16	CTG CAT TAA AAC AAC CAA ACT TG	GAG CTC CAC TTC TTC CAA ACT AG	57
AMS20	TTG AGC AGC AGA ACC AGA C	ATT CGG ACG CAA CAC ATC	59
AMS21	GGT TGT TTC CAC TAC ACT TGA G	CGT CCT TGG TAT TCT TGT GC	56
AMS22	CAC CGT TTC CAT AAT CAA GG	ATT TTT TGG GCA TTG TTG G	58
AMS23	GCT GTT CAC TGG TCT ATC TGG	ATT CGG TGC TGA TTT TCG	57
AMS25	GAG GGC AGT GTT AGC ATT CC	GCA ACC TTT CCC CGA GAG	61
AMS30	CAC TAA TGG GGT AAA TAA TGT TCT AC	TTG CCT TGA AAT CCA GAC	57

Genetic diversity within groups (Nei’s *h*, Nei 1973) and genetic differentiation between groups (Wrights *F*_ST_, Wright 1951) were calculated using purpose-written Perl scripts. The spatial genetic structure of the accessions was studied with principal component analysis (PCA) performed using the R statistical software (R Development Core Team 2013, version 3.02). The same software was used for analysis of population structure with discriminant analysis of principal components (DAPC) using the *adegenet* package (Jombart et al. 2010). The DAPC analysis was repeated ten times, after which individual runs were merged using the software CLUMPP (v 1.1.2) (Jakobsson and Rosenberg 2007). The obtained clusters were mapped in ArcGIS 10.0 (ESRI 2011).

### Ethnobotanical Survey

An ethnobotanical survey was sent to the donors of the accessions included in the investigation. Since many of the accessions were gathered more than 20 years ago, many donors were no longer alive or their whereabouts could not be traced. This was in particular the case for the Finnish accessions. In total, current addresses of 50 donors could be found and surveys were sent out to these donors. The survey included questions of donor taxon name (shallot or potato onion) as well as local name of the accession, cultivation practices, phenology, history, and use (ESM 2).

## Results

### Morphology

**Fig. 1:**
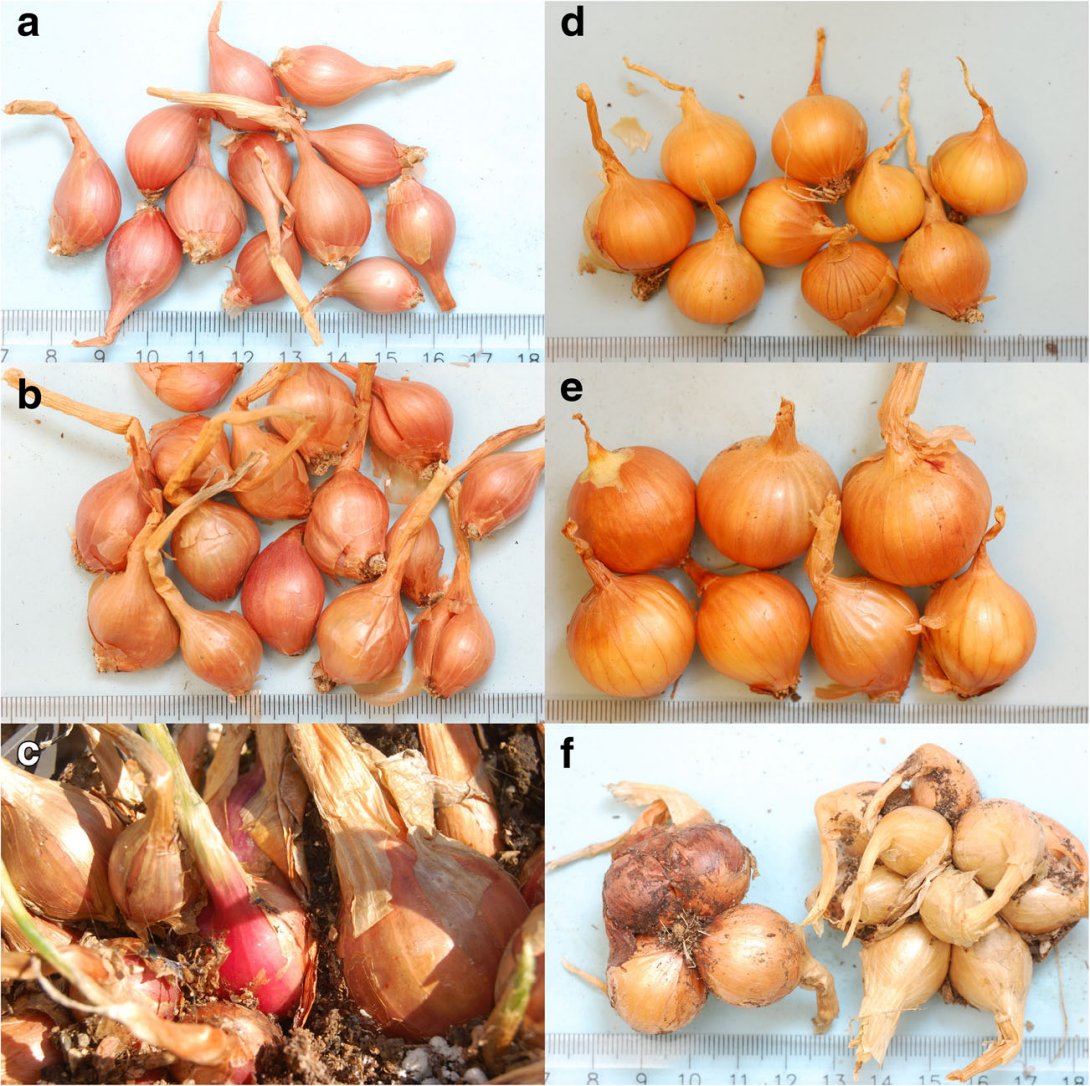
Examples of morphological variation of Nordic multiplying onions. Note accessions with purple skin color (left) and yellow to brown skin color (right). **a** NGB17770 from Norway. **b** SWE104 from Sweden. **c** NGB8315 from Finland. **d** NGB16537 from Denmark. **e** NGB17975 from Norway. **f** NGB17973 from Finland.

Data from cultivation experiments of potato onion at NordGen and the different Nordic programs for biodiversity in cultivated plants were assembled and compared. Many accessions were included in repeated test cultivations, and thus the reliability of the scored characters could be compared. Although there was obvious morphological variation among the material (Fig. 1), characters seemed to be strongly influenced by environment and set onion properties besides genotype. This was especially the case for quantitative traits, such as scores of the size and number of onions produced from each set onion, with large variation among experiments and a strong influence from the size of the set onion. The large variation observed, combined with effects of differences of years and growing locations, limits the possibility to draw conclusions of any quantitative data, and these are therefore not presented. Bulb shape is more of a qualitative character, but was partly differently scored among experiments, where the same accession could produce flattened, round, rhomboid, and high top bulbs. A predominant shape could, however, be assigned to most accessions (ESM 1).

The only character with consistent scoring among experiments was the bulb skin color. This could be classified as either predominantly yellow-brown or red (ESM 1). In all cases where the bulb skin color was red, the flesh color was violet-white, whereas accessions with yellow-brown skin had green-white flesh. Of the 86 accessions scored, nine had red skin and the remaining accessions had yellow-brown skin. The red-skinned accessions were found in all Fennoscandian countries except Denmark.

### Genetic Analysis

We performed molecular genotyping using 12 microsatellite markers. The markers, originally developed for bulb onion, worked well with multiplying onions and yielded relatively consistent results with one or two deviating alleles when the same accessions were subjected to repeated genotyping.

Genetic diversity was calculated for accessions from each country as well as the group of modern varieties (Table 1). The highest diversity was found among the modern varieties, whereas accessions from Georgia were the most homogenous. For the four Fennoscandian countries, only small differences in within-country diversity were found. Genetic differentiation was calculated between groups of accessions from each country (Table 3). Pairwise comparisons between Denmark, Norway, and Sweden resulted in low and non-significant *F*_ST_ values, indicating low differentiation between accessions in these countries. In contrast, Finland, compared to the Scandinavian countries, showed higher and significant *F*_ST_ values. The accessions from Georgia were highly differentiated from all other groups. The modern variety group showed high *F*_ST_ values in comparison to all other groups except the accessions from Finland, suggesting a closer genetic relationship between the Finnish accessions and the modern varieties included in the study.

**Table 3:** *f*_st_ values for pairwise comparisons between countries of origin. average *f*_st_ values across loci.

	Georgia	Finland	Sweden	Norway	Denmark
Finland	0.247***				
Sweden	0.246***	0.115***			
Norway	0.274***	0.110***	0.019		
Denmark	0.292***	0.135***	0.008	0.023	
Modern varieties	0.267***	0.032	0.082***	0.084***	0.109***

***Significant at *P* = 0.001

**Fig. 2:**
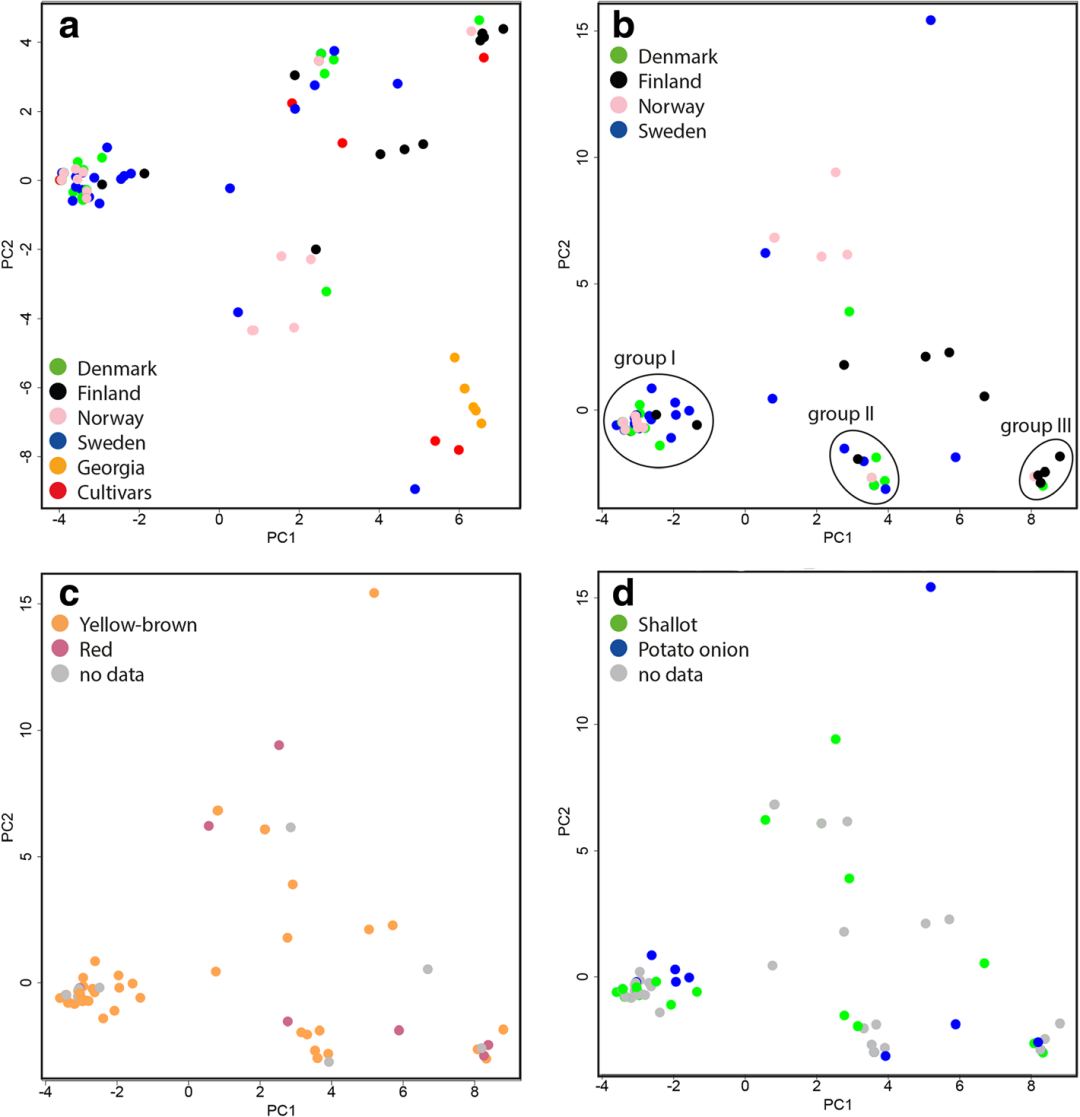
Principal component analyses (PCA) of SSR genotyping of multiplying onions. **a** All accessions, marked by country of origin. **b** All Nordic accessions, marked by country of origin. **c** All Nordic accessions, marked by bulb skin color. **d** All Nordic accessions, marked by name. In **a**, PC1 explains 20.14% of the variation and PC2 10.65%; in **b**–**d**, PC1 explains 23.67% of the variation and PC2 12.83%.

To visualize differentiation of accessions within and between countries and investigate links to morphology and nomenclature, we performed principal component analysis (PCA). This analysis again showed the clear differentiation of the homogenous group of accessions from Georgia from the Fennoscandian material. The modern varieties were well separated from each other and in many cases located away from the Fennoscandian onions, however with notable exceptions (Fig. 2a). Looking only at accessions from the Fennoscandian countries, a large group of genetically similar accessions was identified (Fig. 2b, group I). This group included several accessions from all four countries, in particular Denmark. Two minor groups with genetically similar accessions were also found (Fig. 2b, groups II and III) as well as several accessions that were more genetically unique. We further investigated whether the morphologically stable skin color trait was mirrored in the genetic analyses (Fig. 2c). Although the major cluster only contained yellow-brown skinned accessions, no clear clustering of red and yellow-brown accessions, respectively, was found. Similarly, genetic clustering was not associated with nomenclature, and the same cluster could contain both accessions called “shallot” and those called “potato onion.” On the contrary, the different clusters contained accessions with both names (Fig. 2d).

**Fig. 3:**
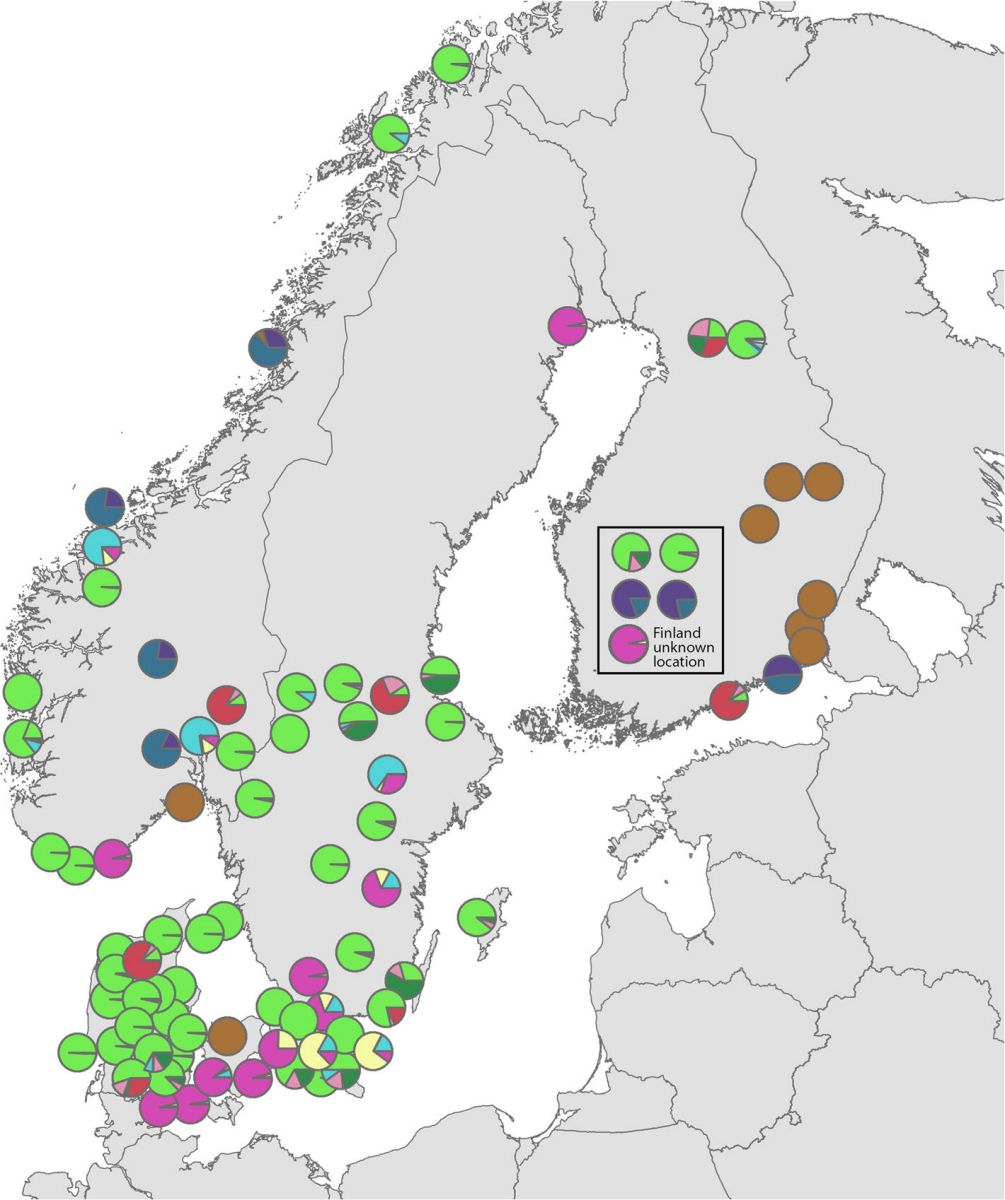
Geographical visualization of DAPC clustering of Nordic multiplying onions. Pie charts show the proportion of designation to each of *N* clusters. Finnish accessions with unknown location of origin are shown in a separate block.

To geographically visualize distribution of genetic variability, we performed discriminant analysis of principal components (DAPC). In the model, we used ten different genetic groups as BIC values formed a plateau around this number (data not shown). The proportion of assignment to each group for each accession was plotted on a map at the location of the origin of the accession (Fig. 3). The most common group (light green) was frequently found in Denmark, especially Jutland, but also in Sweden and along the Norwegian coast. In addition, three of the Finnish accessions primarily belonged to this group. Another group (purple) seems particularly common in eastern Denmark and southernmost Sweden, but single accessions belonging to this group were also found in Norway, northern Sweden, and Finland. The most common Finnish group (brown) was also represented, with one accession in Denmark and one in Norway. The yellow group was only found in a few accessions from southernmost Sweden. Accessions including the dark blue and purple group were found in Norway and Finland, but not in Denmark or southern Sweden. Other minor groups had no obvious geographical distribution. In summary, although some geographical distribution patterns can be seen, many genetic groups are spread over all of Fennoscandia.

Since multiplying onions are mainly vegetatively propagated, a high degree of clonality among accessions was expected. We identified eight genotypes that were shared by two or more accessions. The most common genotype was found in 13 accessions, mainly from Denmark. The two second most common genotypes were each found in five accessions. The first of these two genotypes was represented by accessions from Denmark, Norway, and Sweden, and the second genotype by accessions from Denmark and Sweden and the modern variety ‘Success.’ All the three most common genotypes (comprising 23 accessions) were closely related to each other, differing only in single locus. They belong completely or mainly to the light green genetic group in Fig. 3 and are included in group I in Fig. 2b. The remaining shared genotypes were represented by two or three accessions. Noteworthy are the accessions NGB8315 and NGB17967 from Finland that have identical genotypes to the modern variety ‘Santé.’

### Survey Results

A total number of 24 survey responses were received (48%). The majority of responses were from Denmark and Sweden. The replies are summarized in Table 4, but not all respondents replied to all questions. All respondents from Denmark and Norway responded they knew only “shallot” and therefore could not distinguish between the types “potato onion” and “shallot” (Table 4). Two of the Swedish respondents replied similarly (respondents SWE85 and SWE86) and another two that shallots and potato onion were the same (respondents SWE96 and SWE84). One respondent claimed that potato onion stores better than shallots (respondent SWE87), and another claimed that potato onion was more circular in shape and tasted better than shallots (respondent SWE89).

**Table 4:** Summarized information from the ethnobotanical survey.

Trait	Number of responses	Comments
Donor taxon name		
Shallots (S)	16	Denmark and Norway: all S, Sweden: 6/14 = S
Potato onion (P)	5	Denmark and Norway: no P, Sweden: 5/14 = P
Other names	3	All three in Sweden
No answer	2	
Size of set onion		
Small	11	
Large	1	
Other criteria	9	Medium-sized mentioned by five of the nine respondents
No answer	5	
Flowering		
No	12	
Yes, sometimes	8	Neither of the respondents have used the seeds for seeding
No answer	6	
Keeping ability		
Until spring	7	
Approx. 1 year	6	
More than 1 year	4	
No answer	7	

Regarding cultivation and phenology, most respondents used small- or medium-sized bulbs for cultivation (Table 4). Cultivation was done by planting in rows (at various distances) in spring. Harvesting was done in late summer when leaves were dry. No clear patterns were detected between donor taxon name and the size of bulbs used for cultivation or other cultivation practices. More than half of the respondents had never observed flowering plants among the onions (Table 4). Of the respondents that confirmed flowering, most claimed that this was occurring only at low frequency. One of the respondents (SWE82) reported that 1–2% of the onions planted flowered. Another respondent claimed that flowering occurred after early planting (respondent SWE89). No clear relationship was detected between donor taxon name and flowering. None of the respondents had ever obtained or used true seed for propagation.

The keeping ability varied from over the winter until more than 2 years (Table 4). Dry and cool storage conditions were mentioned as important by most respondents. A proper outdoor curing period prior to storing was mentioned by a few respondents as important to avoid decay (SWE82, SWE106). Storing on nets or in thin layers in boxes was reported (respondents NGB16552 and 16549). Others mention storing in the boiler room or cellar (SWE58, SWE89, SWE96).

For many of the respondents, the histories of the plants were highlighted, in some cases with a long story dating back at least 50 years. Often, the onions were given from one generation to the next within a family. When details were present, a mother to daughter line could often be detected (in Scandinavian “mormor”). Only in one case was a male mentioned (respondent NGB16555). The onions were used in a lot of different dishes and no clear pattern or country differences were detected.

## Discussion

Although no common cultivation experiment with all accessions included has been performed, the comparison of data clearly showed the difficulty in performing characterization of accessions based on morphological characters. The only character that showed a consistent scoring among experiments was red skin color, co-occurring with violet flesh color. Accessions with these traits were, however, not apparently genetically related (Fig. 2c). Other morphological characters as well as agronomic performance seem to be strongly influenced by environmental factors, properties of the set onions, and possibly genotype-environment interactions. We chose not to report quantitative data as these would have required growth trials of all accessions in parallel to be comparable. Repeated experiments in multiple years and locations will be required to evaluate the effects of genotype, environment, and interactions.

A number of potential clones, i.e., accessions with identical genotypes, were identified. It must be noted that the number of genetic markers is small and that more markers could putatively reveal genetic differences between accessions here considered as potential clones. Conversely, there is also a small risk of genotyping errors due to stuttering in the microsatellite analysis that, for example, make differentiation between homozygous and heterozygous loci difficult (Shinde et al. 2003). Likewise, although the proportion of missing data was very low (4.7% across genotyped loci), the sporadic missing data, so-called allelic dropout, could mean accessions have been identified as identical or more similar than they in fact are. Taken together, this means the number of identical clones could be either higher or lower than the numbers presented above. In spite of these concerns, we can with confidence identify a high degree of clonality among the Fennoscandian multiplying onions. This is expected as this plant material has historically and at present only been propagated vegetatively (Nygårds and Leino 2013). The survey results confirmed that flowering is very rare and that seed propagation (i.e., sexual reproduction) is never used in Nordic cultivation of multiplying onions.

The high degree of clonality differs from results obtained in other vegetatively propagated crops in Fennoscandia. In hops (*Humulus lupulus* L.), no identical clones were found when comparing accessions from different Swedish locations (Strese et al. 2014). Likewise, in horseradish (*Armoracia rusticana* G. Gaertn., B. Mey. & Scherb.) collected from Nordic gardens, low clonality was found (Wedelsbäck Bladh et al. 2014). For these crops, repeated introductions over a long period of time and possible sexual reproduction were suggested. The contrasting results obtained for Fennoscandian multiplying onions would suggest that onions have instead been introduced at only a few occasions and then multiplied and spread within Fennoscandia.

Three genetic groups, each including several accessions, could each be linked to modern varieties. The first and largest group of accessions being genetically identical or nearly identical (group I in Fig. 2b, light green in Fig. 3) is closely related to the modern variety ‘Success.’ This type was dominating in Denmark, but was also common in Norway and occurred also in Finland and Sweden. A second group of genetically similar accessions includes the modern variety ‘Santé’ (group III in Fig. 2b, brown in Fig. 3). These accessions were common in Finland, but were also present in Denmark and Norway. A third group (group II in Fig. 2a, purple in Fig. 3) contained several accessions from southeast Denmark and occasional accessions from Sweden, Finland, and Norway as well as the modern variety ‘Red Sun.’ Some of the Finnish accessions with unknown origin (boxed in Fig. 3) might also be of modern variety or breeding material origin according to passport data. Whether these Fennoscandian landrace accessions, being genetically identical to commercial cultivars, actually originate from commercially marketed set onions is not clear. Another possibility is that these accessions are genuine landraces and that the same clones have been gathered and commercialized by seed companies. Although shallot and potato onion have been marketed in Fennoscandia since the 1850s, cultivar names are rarely indicated (Börjeson 2015). Whatever the history of these accessions and their commercial counterparts, the clones or types have apparently been highly appreciated and set onions have been shared and spread widely.

Besides the major groups, the genetic analysis identified a handful of genetically unique clones represented by one or only a few accessions. The clustering of genetically similar or identical accessions in narrow geographical areas is likely explained by sharing of set onions from gardener to gardener within families or in local networks. The exchange of plant material is also confirmed in many of the survey answers where the history of specific accessions can be traced back to several different growers many decades, sometimes up to 100 years ago. None of the respondents reported an origin in commercial purchase of set onions. This does not rule out that some of the multiplying onions have an origin from commercial retailers, but clearly the onions have been maintained and spread by household gardeners for several decades. In the cases where identical clones occur geographically widespread, an involvement from retailers of plant material could be assumed to be more likely. However, several survey respondents said that set onions had been brought along when the respondents or their ancestors had moved within or between the Fennoscandian countries.

Looking at a wider geographical pattern, accessions from the Scandinavian countries—Sweden, Norway, and Denmark—share substantial genetic identity, although some genetic groups are country-specific. In Finland, diversity is higher and the country-wise *F*_ST_ comparisons separate the Finland from the other counties. One reason, which also explains a lower *F*_ST_ in comparison to modern varieties, is a high number of accessions identical or similar to the variety Santé. The results may suggest a different route of introduction of multiplying onions to Finland. Historical sources from the early 20th century mention that set onions were regularly imported to Finland from southern Russia where the onions were propagated from true seed (Lundén 1920). The accessions from Georgia were, however, with the exception of a Swedish accession, not very closely related genetically to the Nordic material. Analyses of more onions from Russia and possibly also the Baltic states could answer whether Finnish multiplying onions have closer genetic resemblance to eastern European onions, which would suggest an Eastern European introduction route.

A long-standing question has been the differences and similarities between shallot and potato onion. Among our material, we could not observe any morphological features that characterized accessions denoted either as shallots or potato onions by the home growers. For example, the morphological character giving the most consistent scoring, color, was clearly not associated with naming. Other morphological characters were very unstable, but could possibly be identified in test cultivations of all accessions at the same location and with set onions of homogenous size. Accessions named shallots and potato onions did not group genetically in any way either (Fig. 2d). In fact, genetically identical accessions occur where one accession is denoted potato onion and the other shallot. The only clear pattern among Fennoscandian multiplying onions is linguistic. In Norwegian and Danish, all multiplying onions are called shallots. In Swedish and Finnish, the names shallot and potato onion are alternatively used. The lack of morphological or genetic distinguishing features contradicts historical literature where shallot and potato onion are clearly separated (Lyttkens 1904 and references therein). One explanation might be that historical sources referring to shallot might actually refer to gray shallot, nowadays identifying as another species, *Allium oschaninii* O. Fedtsch (Maaß 1996). There is, however, no evidence of any actual cultivation of gray shallot in the Nordic countries.

Multiplying onion has long been neglected by commercial growers in the Nordic countries, but instead maintained in household gardens. This is now beginning to change with an emerging interest among chefs for locally produced food with cultural traditions. In addition, multiplying onion is highly appreciated for excellent taste. Thus, an increased commercialization of multiplying onion could be expected. Based on our data, accessions with contrasting genotypes could be chosen for cultivation experiments and identification of suitable accessions for different purposes.

## Electronic Supplementary Material

ESM 1. Full list of accessions with morphological scores for the Nordic accessions. (XLSX 18 kb)

ESM 2. Ethnobotanical survey sent out to donors from Denmark, Norway, and Sweden. (DOCX 19 kb)
