# Supplementary material for: Patterns of Exchange of Multiplying Onion (Allium cepa L. Aggregatum-Group) in Fennoscandian Home Gardens
Source: Econ Bot. 2018 Oct 22;72(3):346–56. doi: 10.1007/s12231-018-9426-2 (PMC6267692; doi:10.1007/s12231-018-9426-2)
Supplement: Supplementary file 2 — Ethnobotanical survey sent out to donors from Denmark, Norway, and Sweden. (DOCX 19 kb) [file 12231_2018_9426_MOESM2_ESM.docx]

Hello,

We write to you because you have donated seed onions to the national program for conservation of cultivated plants. The plant material is conserved within the national programs and we are now carrying out a comparative investigation of onion from the different Nordic countries. The project is being carried out by the Swedish museum of cultural history together with Linköping University and NordGen in Alnarp.

We would be very grateful if you could answer a few short questions about the onion you have cultivated and donated for conservation. What you can tell about your onion is an important part of the history of the donated material, which is also important to document and preserve. Please send your answer in the enclosed envelope to Matti Leino, Nordiska museet, Box 27820, 115 93, Stockholm.

Many thanks for your participation

Matti Leino, Swedish museum of cultural history, Stockholm

Jenny Hagenblad, Linköping University

Svein Solberg, NordGen, Alnarp

1. What do you call the tyoe of onion you have donated?

[ ] potato onion [ ] shalot [ ] other name………………………………………………………………………………

2. Are you aware of both potato onion and shallot and if so what is the difference between the two?

……………………………………………………………………………………………………………………………………………………………………….

……………………………………………………………………………………………………………………………………………………………………….

3. Do you have your own name on your onion or do you have a variety name that you associate it with?

……………………………………………………………………………………………………………………………………………………………………….

4. Have you used any particular selection criterion when you have cultivated your onion?

[ ] large onions [ ] small onions [ ] other criterion…………………………………………………

5. Briefly describe how you cultivate the onion....................................................................................................

…………………………………………………………………………………………………………………………………………………………………………

…………………………………………………………………………………………………………………………………………………………………………

…………………………………………………………………………………………………………………………………………………………………………

…………………………………………………………………………………………………………………………………………………………………………

…………………………………………………………………………………………………………………………………………………………………………

6. Does it happen that the onion flowers? Will the seed ripen so that it can be sown?.......................................

…………………………………………………………………………………………………………………………………………………………………………

…………………………………………………………………………………………………………………………………………………………………………

7. How long can the onion be saved after harvest and how do you keep it?........................................................

…………………………………………………………………………………………………………………………………………………………………………

…………………………………………………………………………………………………………………………………………………………………………

8. Do you use the onion for cooking? How and for what kind of dishes?

…………………………………………………………………………………………………………………………………………………………………………

…………………………………………………………………………………………………………………………………………………………………………

…………………………………………………………………………………………………………………………………………………………………………

…………………………………………………………………………………………………………………………………………………………………………

9. Where did you get the onion? What do you know about its history?

…………………………………………………………………………………………………………………………………………………………………………

…………………………………………………………………………………………………………………………………………………………………………

…………………………………………………………………………………………………………………………………………………………………………

………………………………………………………………………………………………………………………………………………………………………...

…………………………………………………………………………………………………………………………………………………………………………
